# Silencing of long non-coding RNA KCNQ1OT1 alleviates LPS-induced lung injury by regulating the miR-370-3p/FOXM1 axis in childhood pneumonia

**DOI:** 10.1186/s12890-021-01609-0

**Published:** 2021-07-23

**Authors:** Ping Wang, Haitao Zhang, Weiqing Zhao, Nini Dai

**Affiliations:** 1 Department of Pediatrics I, The People’s Hospital of Shouguang, No. 43, Jiankang Street, Shouguang City, 262700 Shandong Province China; 2 Department of Digestive Internal Medicine, Qingdao Hospital of Traditional Chinese Medicine (Qingdao Hiser Hospital), No. 4, Renmin Road, Shibei District, Qingdao City, 266033 Shandong Province China; 3 Department of Pediatrics I, Qingdao Hospital of Traditional Chinese Medicine (Qingdao Hiser Hospital), No. 4, Renmin Road, Shibei District, Qingdao City, 266033 Shandong Province China

**Keywords:** Pneumonia, Long non-coding RNA, KCNQ1 overlapping transcript 1, miR-370-3p, Forkhead box protein M1

## Abstract

**Purpose:**

Long non-coding RNAs (lncRNAs) play important roles in the development of pneumonia. We aimed to explore the role of the lncRNA KCNQ1OT1 in pneumonia and its underlying mechanisms.

**Methods:**

The expression of KCNQ1OT1, FOXM1, and miR-370-3p was detected in the serum of 24 children with pneumonia and in 24 healthy controls. Normal human embryonic lung-derived diploid fibroblasts (WI-38 cells) were stimulated with LPS (10 μg/mL) to simulate the cellular model of pneumonia, and cell viability, apoptosis, and inflammation were analysed. Dual luciferase reporter and/or RNA binding protein immunoprecipitation assays were performed to test the relationship between miR-370-3p and KCNQ1OT1/FOXM1. Mice were intratracheally administered LPS (5 mg/kg) to induce an in vivo model of pneumonia, and pathological injury and inflammation were analysed.

**Results:**

The expression of KCNQ1OT1 and FOXM1 was up-regulated, and miR-370-3p was down-regulated in the serum of children with pneumonia, LPS-treated WI-38 cells, and in lung tissues of LPS-treated mice. Silencing of KCNQ1OT1 or overexpression of miR-370-3p suppressed cell apoptosis and inflammation and facilitated cell viability in LPS-treated WI-38 cells. KCNQ1OT1 directly targets miR-370-3p and negatively regulates its expression. FOXM1 was targeted by miR-370-3p and negatively modulated by miR-370-3p. In addition, silencing of KCNQ1OT1 mitigated LPS-induced lung injury and inflammation in mice. The protective effects of KCNQ1OT1 silencing in LPS-treated WI-38 cells and mice were reversed by silencing of miR-370-3p or overexpression of FOXM1.

**Conclusion:**

Silencing of KCNQ1OT1 alleviates LPS-induced lung injury by regulating the miR-370-3p/FOXM1 axis in pneumonia.

**Supplementary Information:**

The online version contains supplementary material available at 10.1186/s12890-021-01609-0.

## Introduction

Pneumonia is a class of inflammatory diseases of the lung that mainly manifests as fever, dyspnoea, chills, chest pain, and cough [1, 2]. The most frequent complication of pneumonia is pleural effusion, which may lead to infectious pleurisy [3]. Currently, the management of pneumonia mainly includes oxygen, antibiotics, supportive therapy, and assisted ventilation [4, 5]. Despite the fact that great progress has been achieved in the treatment of pneumonia, its therapeutic effect is still far from satisfactory [6, 7]. In addition, pneumonia can be triggered by infection with diverse pathogens, such as bacteria, fungi, and viruses [8, 9]. Pneumonia caused by some pathogens, such as *Staphylococcus aureus* (bacteria) [10, 11], *Fusarium* (fungi) [12], and novel coronaviruses (viruses) [13], are difficult to treat. Therefore, it is important to identify effective therapeutic targets to improve pneumonia.

Long non-coding RNAs (lncRNAs), a set of non-coding RNAs, possess a molecular length of longer than 200 nucleotides and cannot encode proteins [14, 15]. With increasing knowledge of lncRNAs, there is growing interest in the role of lncRNAs in pneumonia [16–18]. Zhou et al. reported that down-regulation of lncRNA SNHG16 enhances cell viability and reduces the levels of inflammatory factors as well as cell apoptosis in a cell model of pneumonia [16]. Zhang et al. showed that the silencing of the lncRNA XIST dramatically mitigates cell injury by facilitating cell viability and restraining inflammation as well as apoptosis in a cell model of pneumonia [17]. Nong et al. showed that knockdown of lncRNA NEAT1 represses cell apoptosis, decreases inflammatory cytokine levels, and promotes cell viability in a cell model of pneumonia [18].

The lncRNA KCNQ1 overlapping transcript 1 (KCNQ1OT1), a chromatin regulatory RNA, is located on the human KCNQ1 locus with a length of 91 kb [19, 20]. KCNQ1OT1 is a crucial modulator involved in the pathological processes of several pulmonary diseases [20, 21]. A study by Jiang et al. reported that silencing of KCNQ1OT1 restrains the inflammatory response in LPS-induced mouse models of acute respiratory distress syndrome (ARDS) [21]. Another study by Kang et al. revealed that down-regulation of KCNQ1OT1 promotes cell apoptosis and suppresses cell viability in non-small cell lung cancer (NSCLC) in vitro [20]. However, little is currently known regarding the specific role and underlying mechanism of KCNQ1OT1 in pneumonia.

LncRNAs exert roles by competitively bind to microRNAs (miRNAs) [22]. miRNAs are a class of short ncRNAs with approximately 19 to 24 nucleotides, which contribute to inhibiting translation or degrading mRNA by binding to the 3-UTR of mRNAs [23, 24]. Many studies have demonstrated the potential functions of miRNAs in regulating the progression of pneumonia [25, 26]. Zhang et al. reported that overexpression of miR-146b reduces the inflammatory response as well as cell apoptosis and enhances cell viability in an LPS-induced cell model of pneumonia [25]. Quan et al. indicated that increasing miR-141 reduces cell apoptosis and the production of pro-inflammatory factors and facilitates cell viability in an LPS-induced cell model of pneumonia [26]. In particular, miR-370-3p has attracted considerable attention because of its pivotal role in pneumonia [17, 27]. MiR-370-3p promotes cell viability and represses apoptosis and inflammation in an LPS-induced cell model of pneumonia [27]. Suppression of miR-370-3p eliminates the promoting impact of lncRNA XIST knockdown on cell viability as well as the inhibitory effects of XIST knockdown on the inflammation response and apoptosis in an LPS-induced cell model of pneumonia [17]. Nevertheless, the relationship between lncRNA KCNQ1OT1 and miR-370-3p has not yet been completely expounded in pneumonia.

In the current study, the role of lncRNA KCNQ1OT1 was explored in LPS-induced cellular and mouse models of pneumonia. The downstream mechanisms of KCNQ1OT1 were further verified. We offer the first evidence for the regulatory role of KCNQ1OT1 via regulation of the miR-370-3p/forkhead box protein M1 (FOXM1) axis in pneumonia.

## Methods

### Patients and blood collection

A total of 24 children (12 male and 12 female; mean age ± standard deviation, 12.4 ± 1.18 years; age range, 8–14) with pneumonia were enrolled in the current study. Simultaneously, 24 healthy children with matched gender and age (mean age ± standard deviation, 10.3 ± 2.24 years; age range, 7–14) were selected as the control. The diagnosis of pneumonia was implemented in the light of the World Health Organization acute respiratory infection guidelines [28]. None of the patients received any treatment prior to blood collection, and patients with other complications were excluded. Peripheral venous blood (5 mL) was obtained from all participants. This study was approved by the ethics committee of the People's Hospital of Shouguang (No. 20200905), and the legal guardians of all participants provided written informed consent.

### Cell culture and treatment

Normal human embryonic lung-derived diploid fibroblasts (WI-38 cells) were purchased from the American Type Culture Collection (ATCC; Manassas, VA, USA). WI-38 cells were cultured in Dulbecco’s modified Eagle’s medium containing 10% foetal bovine serum, 100 µg/mL streptomycin, and 100 U/mL penicillin. All cells were maintained at 37 °C in a humidified atmosphere containing 5% CO_2_. To simulate pneumonia-related cell injury in vitro, WI-38 cells were stimulated with LPS (10 μg/mL) for 12 h [29], and cells without LPS treatment served as the control.

### Cell transfection

Small interfering (si)-KCNQ1OT1, si-negative control (NC), miR-370-3p mimics, mimics-NC, miR-370-3p inhibitor, inhibitor-NC, pcDNA-FOXM1, and pcDNA-NC were purchased from RiboBio (Guangzhou, China). The above factors were transfected into WI-38 cells using Lipofectamine 3000 (Invitrogen, Carlsbad, CA, USA) for 48 h in accordance with the user guide.

### Quantitative real-time polymerase chain reaction (qRT-PCR)

TRIzol reagent (Invitrogen) was used to isolate total RNA from WI-38 cells and serum samples. A PrimeScript RT reagent Kit (TaKaRa, Dalian, China) was used to generate complementary DNA (cDNA). qRT-PCR was performed using SYBR Green qPCR SuperMix (Invitrogen). Primers purchased from TaKaRa are listed in Table 1. The PCR amplification programme was as follows: 94 °C for 10 min, followed by 40 cycles at 94 °C for 15 s, 56 °C for 30 s, 72 °C for 1 min, and 72 °C for 10 min. Relative expression levels of KCNQ1OT1, FOXM1, and miR-370-3p were calculated using the 2^−ΔΔCt^ method. KCNQ1OT1 and FOXM1 were normalised to β-actin, and miR-370-3p was normalised to U6.

**Table 1:** Primers for real-time polymerase chain reaction (qRT-PCR) in present study

Gene	Forward	Reverse
KCNQ1OT1	5′-GCACTCTGGGTCCTGTTCTC-3′	5′-CACTTCCCTGCCTCCTACAC-3′
MiR-370-3p	5′-TGTAACCAGAGAGCGGGATGT-3′	5′-TTTTGGCATAACTAAGGCCGAA-3′
FOXM1	5′-ATACGTGGATTGAGGACCACT-3′	5′-TCCAATGTCAAGTAGCGGTTG-3′
U6	5′-CTCGCTTCGGCAGCACATATACT-3′	5′-ACGCTTCACGAATTTGCGTGTC-3′
β-actin	5′-GCCTTCCTTCTTGGGTAT-3′	5′-GGCATAGAGGTCTTTACGG-3′

### Dual luciferase reporter (DLR) assay

The 3'-UTR portion of KCNQ1OT1 or FOXM1 carrying potential binding sites for miR-370-3p was introduced into a psiCHECK-2 vector (Promega, Madison, WI, USA) to construct the KCNQ1OT1 wt vector or FOXM1 wt vector. Analogously, the 3'-UTR fragment of KCNQ1OT1 or FOXM1, including mutated binding sites within the complementary sequences for miR-370-3p, was cloned into a psiCHECK-2 vector (Promega) to generate the KCNQ1OT1 mut vector or FOXM1 mut vector. Subsequently, WI-38 cells were co-transfected with one of the above vectors along with miR-370-3p mimics or mimics-NC using Lipofectamine 3000 (Invitrogen). Relative luciferase activity at 24 h post-transfection was detected using a luciferase reporter assay system (Promega).

### RNA binding protein immunoprecipitation (RIP) assay

The RIP assay was performed using an EZ-Magna RIP kit (Millipore, Billerica, MA, USA). Briefly, WI-38 cells were transfected with mimics-NC or miR-370-3p mimics for 48 h and then lysed in complete RIP lysis buffer. The cell extracts (100 μL) were incubated with RIP buffer containing magnetic beads conjugated with human anti-Ago2 antibody (a core protein of RNA-induced silencing complex that binds to miRNAs and target mRNAs) (Abcam, Cambridge, MA, USA) or mouse IgG (Abcam) (control). The immunoprecipitated RNAs were purified and used for qRT-PCR to detect the expression of FOXM1.

### 3-(4, 5-Dimethyl-2-Thiazolyl)-2, 5-Diphenyl-2-H-Tetrazolium Bromide (MTT) assay

WI-38 cells were plated in 96-well plates (5 × 10^3^ cells/well). After incubation for 48 h, each well was supplemented with 20 μL MTT (Sigma-Aldrich, St. Louis, MO, USA) and incubated at 37 °C for 4 h. Subsequently, dimethyl sulfoxide (150 μL) was added and mixed for 10 min. The optical density of each well at 490 nm was measured using a microplate reader (Bio-Rad, Hercules, CA, USA).

### Evaluation of cell apoptosis

Cell apoptosis was assessed using a fluorescein isothiocyanate (FITC)-conjugated Annexin V and propidium iodide (PI) kit (BD Biosciences, San Diego, CA, USA). Briefly, WI-38 cells were washed with phosphate-buffered saline and stained with Annexin V-FITC (5 μL) and PI (5 μL) for 30 min at 25 °C in the dark. Flow cytometry was used to test apoptotic cells, and data were analysed using FlowJo software (Tree Star Inc., Ashland, OR, USA).

### Western blot

Proteins from WI-38 cells were extracted using RIPA lysis buffer (Beyotime, Shanghai, China). Proteins were separated by sodium dodecyl sulphate–polyacrylamide gel electrophoresis gels and transferred to polyvinylidene fluoride membranes. After blocking with 5% bovine serum albumin for 2 h, the membranes were incubated with primary antibodies against FOXM1 (1:1000, ab207298, Abcam, Cambridge, MA, USA) and α-tubulin (1:2000, ab52866, Abcam) at 4 °C overnight. Next, membranes were washed with Tris-buffered saline with Tween 20 and the secondary antibody (1:2000; ab205718, Abcam) was added to cultivate for 1 h. Finally, proteins bands were visualised via a Bio-Rad Gel Doc EZ Imager (Bio-Rad). Relative protein expression of FOXM1 normalised to α-tubulin was quantified using a ChemiDoc XRS System (Bio-Rad).

### Establishment of a mouse model of LPS-induced lung injury

Male BALB/c mice (specific pathogen-free, six-week-old, weighing 17–19 g) were purchased from Esebio (Shanghai, China). All mice were housed under conditions of constant temperature and humidity and had free access to food and water. To induce pneumonia-related lung injury in vivo, mice were anaesthetised by intraperitoneal injection of 50 mg/kg pentobarbital sodium then intratracheally administered 5 mg/kg LPS (St. Louis, MO, USA) (dissolved in 50 μL normal saline) [30]. Mice in the control group were administered 50 μL of normal saline. Animal experiments were performed after obtaining approval from the Ethical Committee of the People's Hospital of Shouguang (No. 20200905) in accordance with the Guide for the Care and Use of Laboratory Animals.

### Treatments and sample collection

Recombinant adenoviruses containing short-hairpin RNA KCNQ1OT1 (Ad-sh-KCNQ1OT1), FOXM1 (Ad-FOXM1), empty adenovirus NC (Ad-NC), antagomiR-370-3p, and antagomiR-NC were purchased from Ribobio (Guangzhou, China). The above adenoviruses (100 µL, 1 × 10^8^ pfu/mL) and antagomirs (50 mg/kg body weight) were intravenously injected into the mice two days before model induction. After LPS treatment for three days, all mice were anaesthetised by intraperitoneal injection of 50 mg/kg pentobarbital sodium. Bronchoalveolar lavage fluid (BALF) (1.4 mL) was harvested for enzyme-linked immunosorbent assay (ELISA). Mice were then euthanised by cervical dislocation, and the right lung was excised to measure the lung wet/dry ratio. The left lung was used for haematoxylin–eosin (HE) staining.

### ELISA

The levels of tumour necrosis factor-α (TNF-α), interleukin-6 (IL-6), and IL-1β in the culture supernatants of WI-38 cells and BALB/c mice were measured using specific ELISA kits (R&D Systems China, Shanghai, China). A microplate reader (Molecular Devices, Sunnyvale, CA, USA) was used to examine the optical density at 450 nm.

### HE staining

The right lung tissues were fixed in 4% paraformaldehyde for 24 h, dehydrated in a graded ethanol series, embedded in paraffin, and sliced into five slices. After staining with HE, pathological changes in lung tissues were observed under a light microscope (Olympus, Tokyo, Japan). The injury score was calculated as follows: 0, no damage; l, mild damage; 2, moderate damage; 3, severe damage; and 4, severe damage [31].

### Statistical analysis

All experiments were undertaken independently three times. SPSS version 21.0 (IBM Software, New York, NY, USA) was used for statistical analysis. Data in this study are displayed as the mean ± standard deviation. Differences between two groups were analysed using Student’s t-test. For comparisons among multiple groups, one-way ANOVA with Tukey’s post-hoc test was implemented. *P* < 0.05 indicated statistical significance.

## Results

### Silencing of KCNQ1OT1 facilitated cell viability and suppressed apoptosis and inflammation in LPS-induced WI-38 cells

**Fig. 1:**
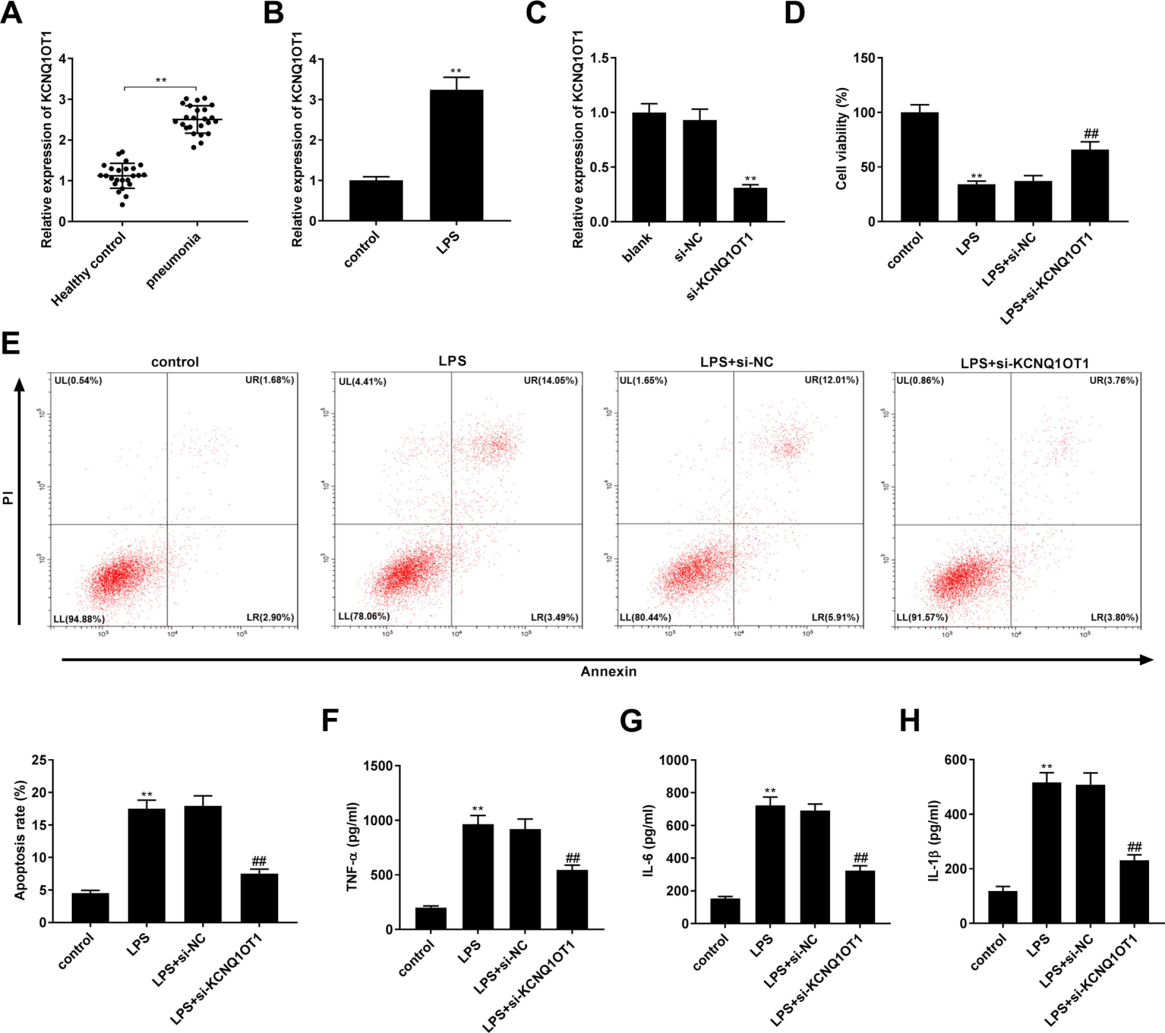
Silencing of KCNQ1OT1 facilitated cell viability and suppressed cell apoptosis and inflammation in LPS-induced WI-38 cells. **A** Relative expression of KCNQ1OT1 was determined by quantitative real-time polymerase chain reaction (qRT-PCR) in the serum of pneumonia patients and healthy controls. ^**^P < 0.01, vs. Healthy control. **B** Relative expression of KCNQ1OT1 was detected by qRT-PCR in WI-38 cells. ^**^P < 0.01, vs. control. **C** Relative expression of KCNQ1OT1 was detected by qRT-PCR in WI-38 cells transfected with small interfering (si)-negative control (NC) or si-KCNQ1OT1. ^**^P < 0.01, vs. si-NC. **D** Cell viability was detected by 3-(4, 5-Dimethyl-2-Thiazolyl)-2, 5-Diphenyl-2-H-Tetrazolium Bromide (MTT) assay in WI-38 cells. **E** The apoptosis rate was detected by flow cytometry in WI-38 cells. Lower left quadrant (LL): viable cells (AnnexinV −/PI −); Upper left quadrant (UL): necrotic cells (AnnexinV −/PI +); Lower right quadrant (LR): early apoptotic cells (AnnexinV +/PI −); Upper right quadrant (UR): late apoptotic cells (AnnexinV +/PI +); The apoptotic cells (%) were calculated as cells in LR + UR. **F** The level of TNF-α was measured by enzyme-linked immunosorbent assay (ELISA) in WI-38 cells. **G** The level of IL-6 was measured by ELISA in WI-38 cells. **H** The level of IL-1β was measured by ELISA in WI-38 cells. (D-H) ^**^P < 0.01, vs. control. ^##^*P* < 0.01, vs. LPS + si-NC

To investigate the role of KCNQ1OT1 in LPS-stimulated WI-38 cells, we initially determined the expression of KCNQ1OT1 by qRT-PCR. The results indicated that the relative expression of KCNQ1OT1 was notably elevated in the serum of patients with pneumonia compared to the serum of healthy individuals (*P* < 0.01, Fig. 1A). Congruously, KCNQ1OT1 in LPS-stimulated WI-38 cells was also observed as opposed to the control (*P* < 0.01, Fig. 1B). Next, we silenced KCNQ1OT1 by transfection with si-KCNQ1OT1. As expected, there was a distinct decrease in the expression of KCNQ1OT1 after transfection of si-KCNQ1OT1 in LPS-induced WI-38 cells (*P* < 0.01, Fig. 1C). Subsequently, we conducted functional experiments to explore the specific function of KCNQ1OT1 in WI-38 cells. The MTT assay demonstrated that LPS treatment triggered a notable decrease in cell viability in WI-38 cells, while down-regulation of KCNQ1OT1 enhanced cell viability in LPS-induced WI-38 cells (all *P* < 0.01, Fig. 1D). The results of flow cytometry showed that treatment with LPS increased the apoptosis rate of WI-38 cells, whereas silencing of KCNQ1OT1 reduced the apoptosis rate in LPS-treated WI-38 cells (all *P* < 0.01, Fig. 1E). In addition, the levels of TNF-α, IL-6, and IL-1β were elevated after treatment with LPS in WI-38 cells, while silencing of KCNQ1OT1 reduced the levels of TNF-α, IL-6, and IL-1β in LPS-induced WI-38 cells (all *P* < 0.01, Fig. 1F–H).

### LncRNA KCNQ1OT1 acted as a sponge for miR-370-3p

**Fig. 2:**
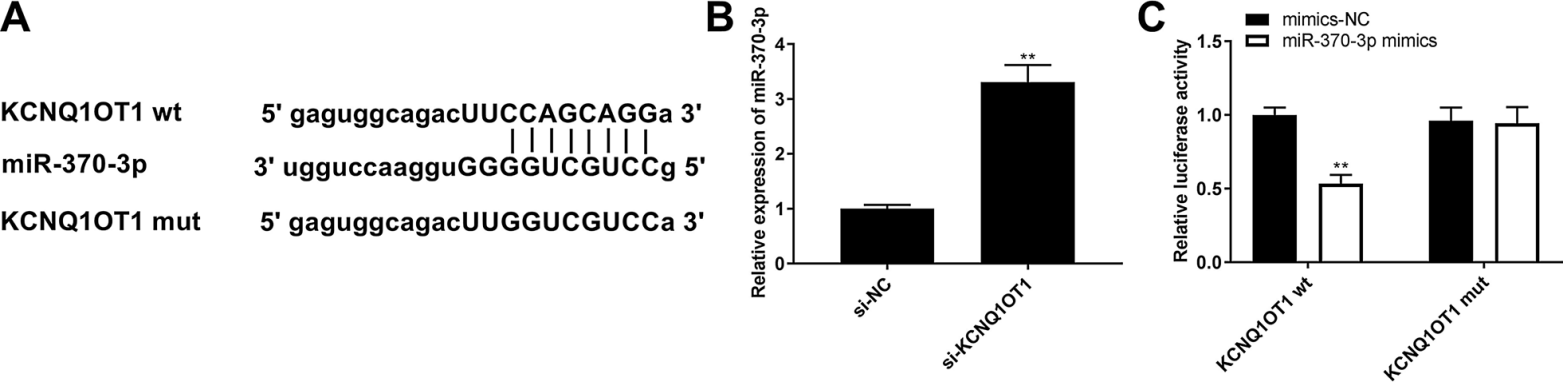
KCNQ1OT1 acted as a sponge for miR-370-3p. **A** A binding site between KCNQ1OT1 and miR-370-3p was predicted by starbase2.0. **B** Relative expression of miR-370-3p in LPS-induced WI-38 cells was detected by quantitative real-time polymerase chain reaction (qRT-PCR). ^**^P < 0.01, vs. small interfering (si)-negative control (NC). **C** The targeting relationship between KCNQ1OT1 and miR-370-3p was validated by dual-luciferase reporter (DLR) assay in LPS-induced WI-38 cells. ^**^P < 0.01. vs. mimics-NC

To clarify the downstream mechanism of KCNQ1OT1, its potential targets were identified using starBase2.0. As shown in Fig. 2A, there was a putative association between KCNQ1OT1 and miR-370-3p. Additionally, the relative expression of miR-370-3p significantly increased with the introduction of si-KCNQ1OT1 in LPS-treated WI-38 cells (*P* < 0.01, Fig. 2B). To verify the interaction between miR-370-3p and KCNQ1OT1, a DLR assay was performed and co-transfected with miR-370-3p mimics and KCNQ1OT1 wt remarkably reduced the relative luciferase activity of WI-38 cells (*P* < 0.01, Fig. 2C), but co-transfection of mimics-NC and KCNQ1OT1 wt failed to change the relative luciferase activity of WI-38 cells (Fig. 2C).

### Overexpression of miR-370-3p enhanced cell viability and repressed apoptosis and inflammation in LPS-induced WI-38 cells

**Fig. 3:**
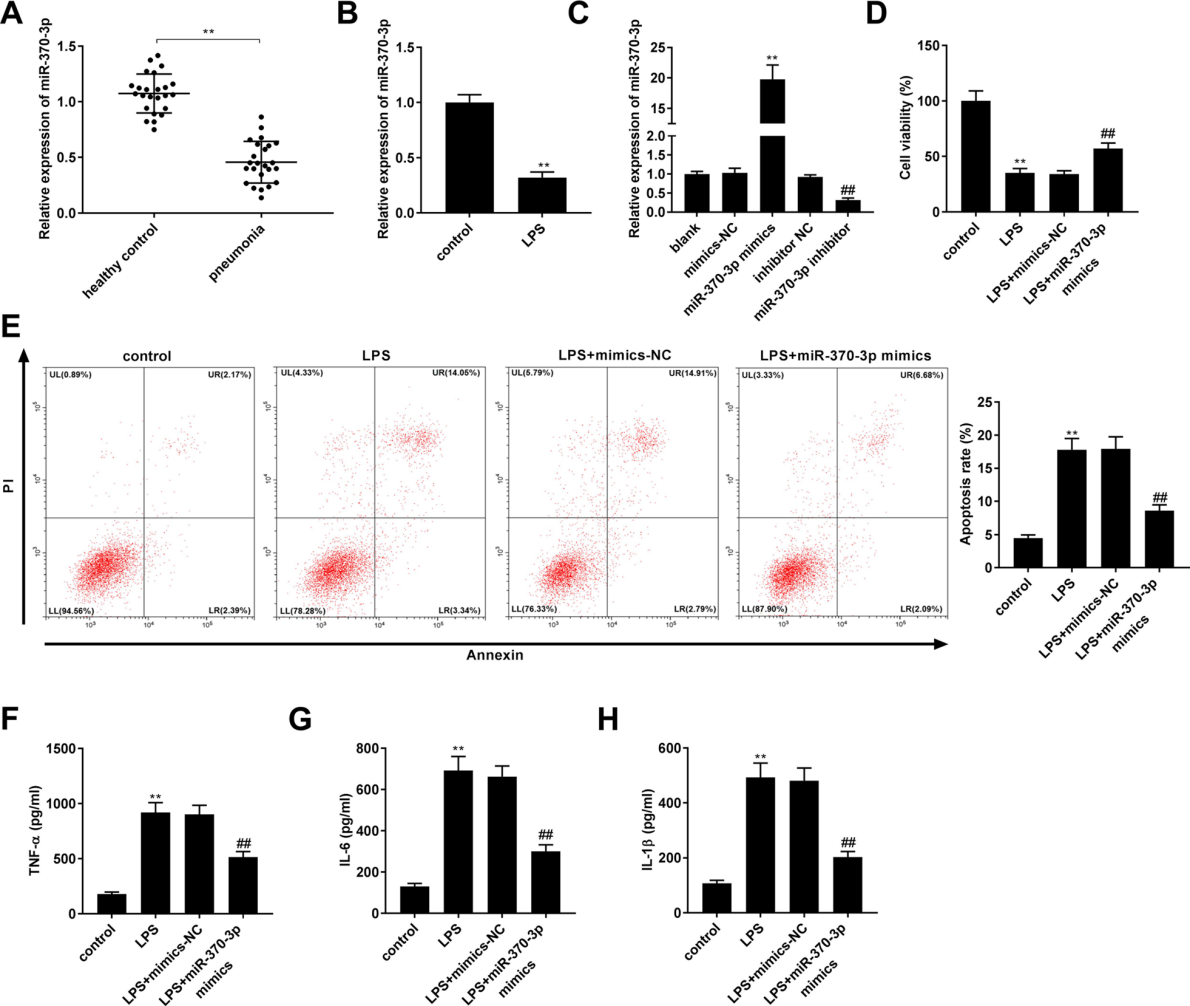
Overexpression of miR-370-3p enhanced cell viability and repressed apoptosis and inflammation in LPS-induced WI-38 cells. **A** Relative expression of miR-370-3p was determined by quantitative real-time polymerase chain reaction (qRT-PCR) in the serum of pneumonia patients and healthy controls. ^**^P < 0.01, vs. healthy control. **B** Relative expression of miR-370-3p was detected by qRT-PCR in WI-38 cells. ^**^P < 0.01, vs. control. **C** The efficiency of miR-370-3p mimics and inhibitor on the expression of miR-370-3p was detected by qRT-PCR in WI-38 cells. ^**^P < 0.01, vs. mimics negative control (mimics-NC). ^##^*P* < 0.01, vs. inhibitor NC. **D** Cell viability was detected by 3-(4, 5-Dimethyl-2-Thiazolyl)-2, 5-Diphenyl-2-H-Tetrazolium Bromide (MTT) assay in LPS-induced WI-38 cells. **E** The apoptosis rate was detected by flow cytometry in LPS-induced WI-38 cells. Lower left quadrant (LL): viable cells (AnnexinV −/PI −); Upper left quadrant (UL): necrotic cells (AnnexinV −/PI +); Lower right quadrant (LR): early apoptotic cells (AnnexinV +/PI−); Upper right quadrant (UR): late apoptotic cells (AnnexinV +/PI +); The apoptotic cells (%) were calculated as cells in LR + UR. **F** The level of TNF-α in LPS-induced WI-38 cells was measured by enzyme-linked immunosorbent assay (ELISA). **G** The level of IL-6 in LPS-induced WI-38 cells was measured by ELISA. **H** The level of IL-1β in LPS-induced WI-38 cells was measured by ELISA. **D**–**H** ^**^P < 0.01, vs. control; ^##^*P* < 0.01, vs. LPS + mimics-NC

Next, we examined the expression and function of miR-370-3p in WI-38 cells. As shown in Fig. 3A, the relative expression of miR-370-3p in the pneumonia group was lower than that in the healthy control group (*P* < 0.01, Fig. 3A). Consistently, the relative expression of miR-370-3p in the LPS group was lower than that in the control group (*P* < 0.01, Fig. 3B). The following gain- and loss-of-function assays revealed that miR-370-3p expression was induced by the addition of miR-370-3p mimics and reduced by the addition of miR-370-3p inhibitor in WI-38 cells (all *P* < 0.01, Fig. 3C). Moreover, up-regulation of miR-370-3p facilitated cell viability and inhibited cell apoptosis in LPS-induced WI-38 cells (all *P* < 0.01, Fig. 3D,E). Levels of IL-6, IL-1β, and TNF-α were reduced by miR-370-3p mimics in LPS-induced WI-38 cells (all *P* < 0.01, Fig. 3F–H).

### miR-370-3p directly targeted FOXM1

**Fig. 4:**
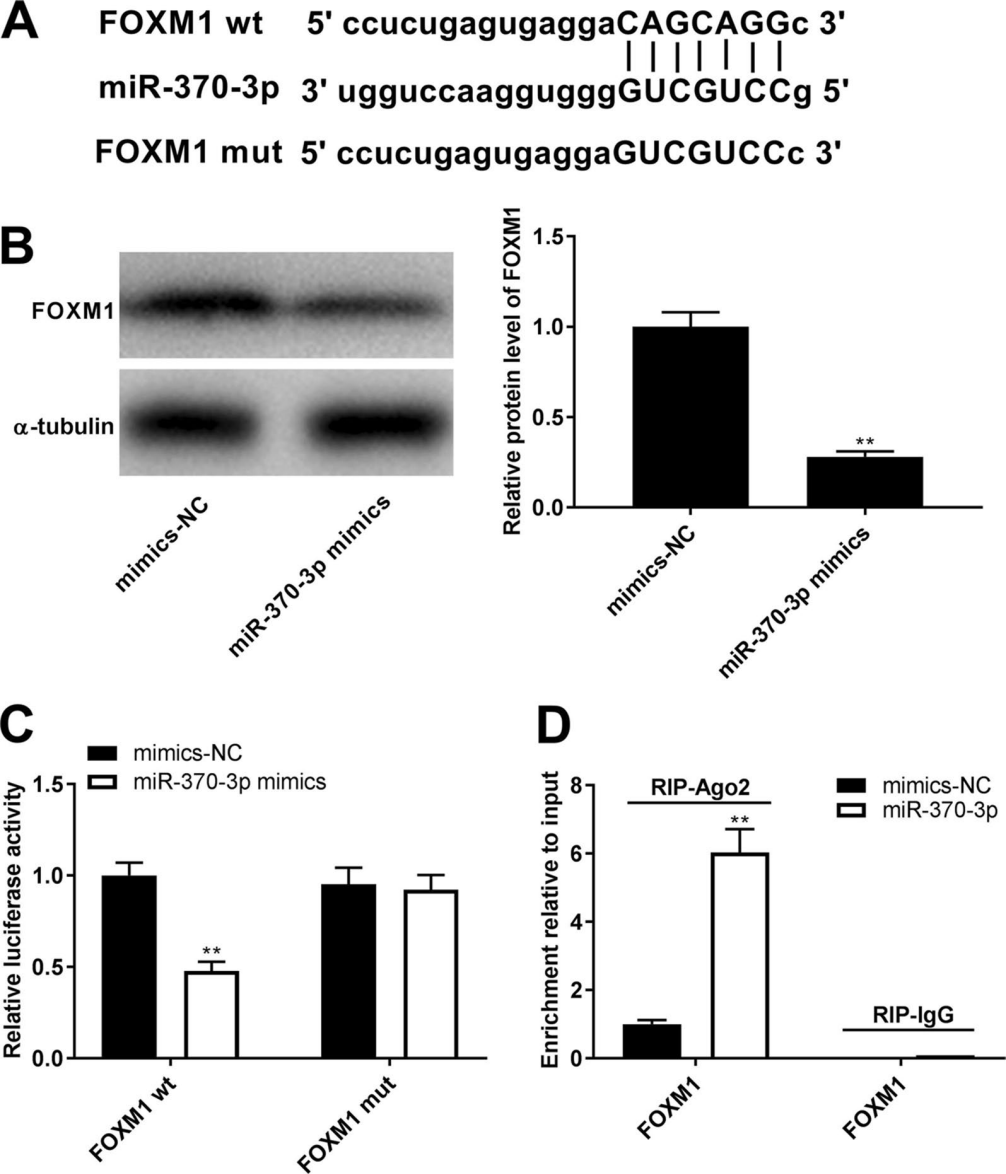
MiR-370-3p directly targeted FOXM1. **A** A binding site between miR-370-3p and FOXM1 was predicted by starbase2.0. **B** Relative protein level of FOXM1 in LPS-induced WI-38 cells was detected by western blot. **C** The targeting relationship between miR-370-3p and FOXM1 was validated by dual-luciferase reporter (DLR) assay in LPS-induced WI-38 cells. **D** The targeting relationship between miR-370-3p and FOXM1 was validated by RNA binding protein immunoprecipitation (RIP) assay in LPS-induced WI-38 cells. (B-D) ^**^P < 0.01, vs. mimics-negative control (NC)

We then examined the mechanism by which miR-370-3p affects WI-38 cells treated with LPS. We observed that the 3'-UTR of FOXM1 was complementary to the seed sequence of miR-370-3p (*P* < 0.01, Fig. 4A). Overexpression of miR-370-3p dramatically reduced the relative protein expression of FOXM1 in LPS-treated WI-38 cells (*P* < 0.01, Fig. 4B). In addition, DLR and RIP assays were employed to further confirm whether FOXM1 was the target of miR-370-3p. The DLR assay showed that up-regulation of miR-370-3p led to a distinct reduction in relative luciferase activity of FOXM1 wt compared with the mimics-NC group (*P* < 0.01, Fig. 4C), but it had no evident impact on the relative luciferase activity of FOXM1 mut (Fig. 4C). The RIP assay showed that the enrichment of FOXM1 was significantly increased in the miR-370-3p group compared to in the mimics-NC group (*P* < 0.01, Fig. 4D).

### Knockdown of KCNQ1OT1 alleviated LPS-induced damage in WI-38 cells by regulating the miR-370-3p/FOXM1 axis

**Fig. 5:**
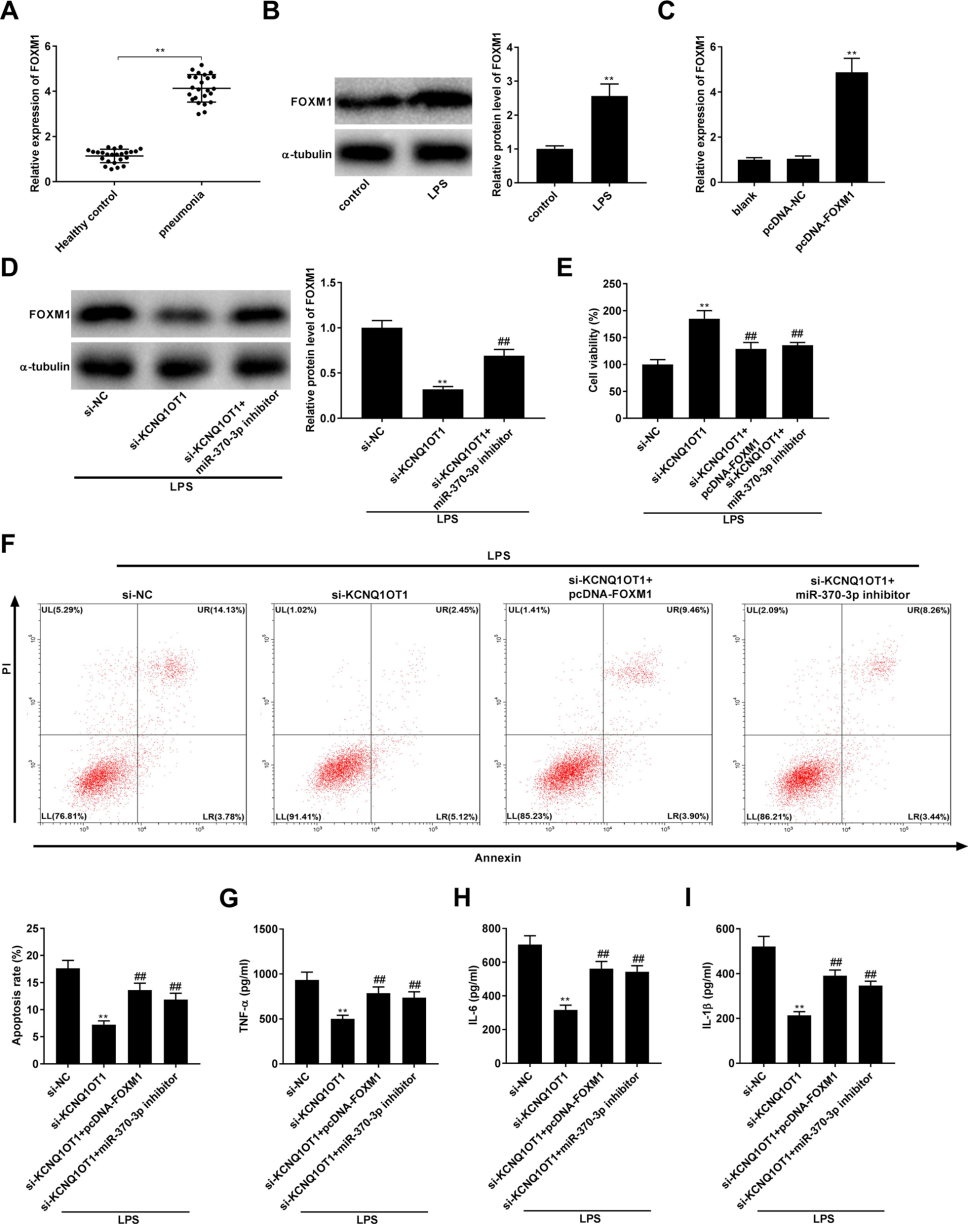
Knockdown of KCNQ1OT1 alleviated LPS-induced damage of WI-38 cells by regulating miR-370-3p/FOXM1 axis. **A** Relative expression of FOXM1 was determined by quantitative real-time polymerase chain reaction (qRT-PCR) in the serum of pneumonia patients and healthy controls. ^**^P < 0.01, vs. Healthy control. **B** Relative protein level of FOXM1 was detected by western blot in WI-38 cells. ^**^P < 0.01, vs. control. **C** The efficiency of pcDNA-FOXM1 on the expression of miR-370-3p was detected by qRT-PCR in WI-38 cells. ^**^P < 0.01, vs. pcDNA-negative control (NC). **D** Relative protein level of FOXM1 was detected by western blot in LPS-induced WI-38 cells. **E** Cell viability was detected by 3-(4, 5-Dimethyl-2-Thiazolyl)-2, 5-Diphenyl-2-H-Tetrazolium Bromide (MTT) assay in LPS-induced WI-38 cells. **F** The apoptosis rate was detected by flow cytometry in LPS-induced WI-38 cells. Lower left quadrant (LL): viable cells (AnnexinV −/PI −); Upper left quadrant (UL): necrotic cells (AnnexinV −/PI +); Lower right quadrant (LR): early apoptotic cells (AnnexinV +/PI −); Upper right quadrant (UR): late apoptotic cells (AnnexinV +/PI +); The apoptotic cells (%) were calculated as cells in LR + UR. **G** The level of TNF-α in LPS-induced WI-38 cells was measured by enzyme-linked immunosorbent assay (ELISA). **H** The level of IL-6 in LPS-induced WI-38 cells was measured by ELISA. **I** The level of IL-1β in LPS-induced WI-38 cells was measured by ELISA. **D**–**I** ^**^P < 0.01, vs. small interfering (si)-NC. ^##^*P* < 0.01, vs. si-KCNQ1OT1

Next, we investigated the relationships between KCNQ1OT1, miR-370-3p, and FOXM1. We first determined that the relative mRNA expression of FOXM1 in the pneumonia group was up-regulated relative to the healthy control group, and the relative protein expression of FOXM1 in the LPS group was also up-regulated relative to that in the control group (all *P* < 0.01, Fig. 5A-5B). The transfection of pcDNA-FOXM1 significantly increased the expression of FOXM1 in WI-38 cells (*P* < 0.01, Fig. 5C). Down-regulation of KCNQ1OT1 decreased the relative protein level of FOXM1, while suppression of miR-370-3p largely eliminated the si-KCNQ1OT1-induced reduction of FOXM1 in LPS-treated WI-38 cells (all *P* < 0.01, Fig. 5D). In addition, we found that the addition of si-KCNQ1OT1 enhanced cell viability and repressed cell apoptosis, while the enhancement impact of si-KCNQ1OT1 on cell viability and the suppressive effect of si-KCNQ1OT1 on cell apoptosis were vastly reversed by the introduction of miR-370-3p inhibitor or pcDNA-FOXM1 in LPS-treated WI-38 cells (all *P* < 0.01, Fig. 5E, F). The levels of IL-1β, TNF-α, and IL-6 were reduced by transfection with si-KCNQ1OT1, whereas these reductions were partly abolished by the introduction of the miR-370-3p inhibitor or pcDNA-FOXM1 in LPS-treated WI-38 cells (all *P* < 0.01, Fig. 5G–I).

### Knockdown of KCNQ1OT1 mitigated LPS-induced lung injury and inflammation in mice by regulating the miR-370-3p/FOXM1 axis

**Fig. 6:**
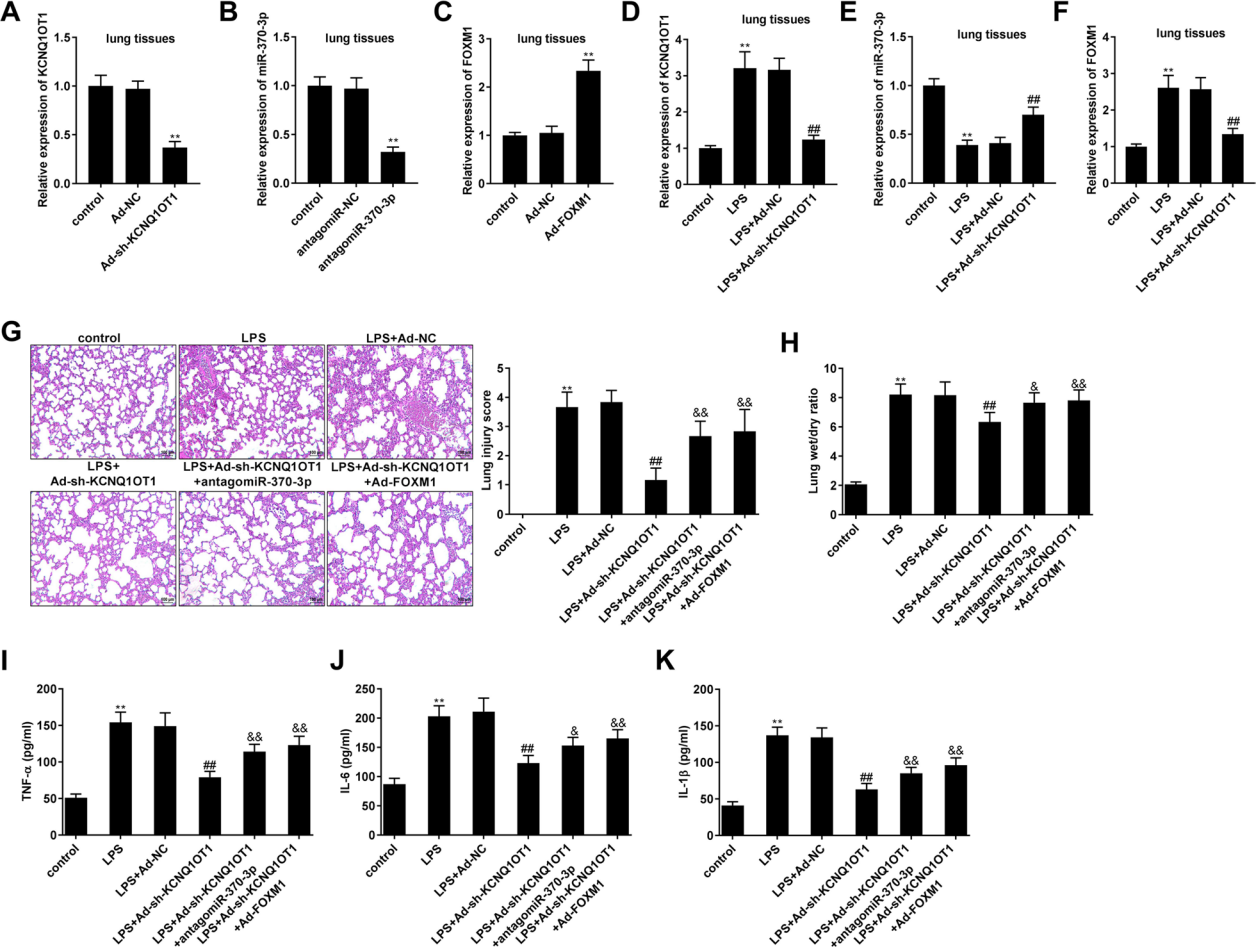
Knockdown of KCNQ1OT1 mitigated LPS-caused lung injury and inflammation in mice through regulating miR-370-3p/FOXM1 axis. **A** The efficiency of Ad-sh-KCNQ1OT1 on the expression of KCNQ1OT1 was detected by quantitative real-time polymerase chain reaction (qRT-PCR) in lung tissues of mice. ^**^P < 0.01, vs. adenovirus negative control (Ad-NC). **B** The efficiency of antagomiR-370-3p on the expression of miR-370-3p was detected by qRT-PCR in lung tissues of mice. ^**^P < 0.01, vs. antagomiR-NC. **C** The efficiency of Ad-FOXM1 on the expression of FOXM1 was detected by qRT-PCR in lung tissues of mice. ^**^P < 0.01, vs. Ad-NC. **D** Relative expression of KCNQ1OT1 was determined by qRT-PCR in LPS-induced mice. **E** Relative expression of miR-370-3p was determined by qRT-PCR in LPS-induced mice. **F** Relative expression of FOXM1 was determined by qRT-PCR in LPS-induced mice. **G** Hematoxylin–eosin (HE) staining of lung tissues in LPS-induced mice and lung injury score (Magnification 400 × , scale bar 50 μm). **H** Lung wet/dry ratio. **I** The level of TNF-α in bronchoalveolar lavage fluid (BALF) was measured by ELISA. **J** The level of IL-6 in BALF was measured by ELISA. **K** The level of IL-1β in BALF was measured by ELISA. **D**–**K** ^**^P < 0.01, vs. control. ^##^*P* < 0.01, vs. LPS + Ad-NC. ^&^*P* < 0.05, ^&&^*P* < 0.01, vs. LPS + Ad-sh-KCNQ1OT1

The regulatory role of the KCNQ1OT1/miR-370-3p/FOXM1 axis in pneumonia was further analysed in a mouse model of LPS-induced lung injury. The injection of Ad-sh-KCNQ1OT1 and antagomiR-370-3p significantly decreased the expression of KCNQ1OT1 and miR-370-3p in lung tissues of mice, respectively (all *P* < 0.01, Fig. 6A, B). The injection of Ad-FOXM1 significantly increased the expression of FOXM1 in lung tissues of mice (*P* < 0.01, Fig. 6C). Silencing of KCNQ1OT1 reversed LPS-induced up-regulation of KCNQ1OT1 and FOXM1 and down-regulation of miR-370-3p in lung tissues of mice (all *P* < 0.01, Fig. 6D–F). Silencing of KCNQ1OT1 also eliminated the LPS-induced increase in lung injury score and lung wet/dry ratio in mice (*P* < 0.01). The mitigation effects of sh-KCNQ1OT1 on lung injury score and lung wet/dry ratio were reversed by silencing of miR-370-3p or overexpression of FOXM1 (all *P* < 0.05, Fig. 6G, H). In addition, silencing of KCNQ1OT1 eliminated the LPS-induced increase in TNF-α, IL-1β, and IL-6 levels in BALF of mice (*P* < 0.01). The inhibitory effect of sh-KCNQ1OT1 on inflammation was reversed by silencing of miR-370-3p or overexpression of FOXM1 (all *P* < 0.05, Fig. 6I–K).

## Discussion

Pneumonia is an acute inflammation of the lower respiratory tract, which is considered the most widespread infectious disease and requires unremitting attention [6, 32]. Up-regulation of lncRNA KCNQ1OT1 is intrinsically relevant to the pathogenesis of pulmonary diseases [21, 33, 34]. Jiang et al. reported that the expression of KCNQ1OT1 in neutrophils of ARDS mice is considerably higher than that in the neutrophils of control mice [21]. Zheng et al. demonstrated that KCNQ1OT1 is highly expressed in NSCLC tissues relative to normal tissues [33]. Ren et al. reported that the expression of KCNQ1OT1 in lung adenocarcinoma tissues was higher than that in control tissues [34]. In line with the expression trend of previous studies, we also observed the up-regulation of KCNQ1OT1 in the serum of patients with pneumonia and LPS-treated WI-38 cells and mice compared with the controls. Therefore, we speculated that KCNQ1OT1 participates in the progression of pneumonia. Pneumonia may be diagnosed by measuring KCNQ1OT1 expression in the serum of patients.

In contrast, accumulating evidence suggests that KCNQ1OT1 plays a pivotal role in the regulation of cell apoptosis, viability, and inflammation of several diseases [35, 36]. For instance, down-regulation of KCNQ1OT1 facilitates cell viability while repressing cell apoptosis and production of inflammatory cytokines in myocardial infarction [35]. Inhibition of KCNQ1OT1 promotes cell viability, reduces secretion of inflammatory factors, and suppresses cell apoptosis in a cell model of myocardial ischaemia/reperfusion injury [36]. Silencing of KCNQ1OT1 promotes apoptosis and suppresses the proliferation of NSCLC cells [20]. Analogous to prior findings, we discovered that knockdown of KCNQ1OT1 suppressed cell apoptosis and the inflammation response, whereas it facilitated cell viability in LPS-treated WI-38 cells. In addition, a previous study confirmed that the down-regulation of KCNQ1OT1 suppresses the inflammatory response by decreasing the levels of TNF-α and IL-6 in a mouse model of ARDS [21]. In this study, KCNQ1OT1 knockdown alleviated LPS-induced lung injury and inflammation in LPS-treated mice. These results indicated that KCNQ1OT1 knockdown alleviated LPS-induced injury both in vitro and in vivo. KCNQ1OT1 may be a potential target for the treatment of pneumonia, showing promising prospects in clinical practice.

To our knowledge, a large number of lncRNAs affect the development of diseases by competitively binding miRNAs to modulate the expression of target genes [37–39]. KCNQ1OT1 directly targets miR-27b-3p [40] and miR-204-5p [20] in NSCLC and targets miR-381-3p in ARDS [21]. We confirmed that lncRNA KCNQ1OT1 directly interacts with miR-370-3p and reverse regulates the expression of miR-370-3p in LPS-induced pneumonia in vitro and in vivo. Furthermore, previous studies have uncovered the down-regulation and crucial impact of miR-370-3p in pneumonia [17, 27]. For example, miR-370-3p is down-regulated in the serum of patients with acute pneumonia and promotes cell viability and represses inflammation and apoptosis in an LPS-induced cell model of pneumonia [27]. Down-regulation of miR-370-3p reverses the promoting impact of lncRNA XIST knockdown on cell viability as well as the suppressive effects of XIST knockdown on the inflammatory response and apoptosis in an LPS-induced cell model of pneumonia [17]. miR-370-3p regulates LPS-induced acute pneumonia in WI-38 cells by targeting TLR4 [41]. In the present study, miR-370-3p was down-regulated in the serum of patients with pneumonia and LPS-treated WI-38 cells and mice, which was consistent with the expression trend of prior findings. We speculated that the expression of miR-370-3p in the serum of patients might be a diagnostic marker for pneumonia. In addition, miR-370-3p inhibited apoptosis, inflammation, and enhanced the viability of LPS-treated WI-38 cells. These results indicated that the up-regulation of miR-370-3p could alleviate LPS-induced injury in WI-38 cells, showing a potential promising strategy in the treatment of pneumonia in clinical practice. More importantly, we discovered that repression of miR-370-3p overturned the protective effects of KCNQ1OT1 silencing in both LPS-treated WI-38 cells and mice. These results indicated that silencing of KCNQ1OT1 protected against LPS-induced injury by sponging miR-370-3p in pneumonia.

FOXM1, a member of the Forkhead box (FOX) family of transcription factors, is expressed in proliferating cells and shares homology in the Winged Helix/Forkhead DNA binding domain [42, 43]. Up-regulation of FOXM1 has been unveiled in pulmonary allergen sensitisation-induced airway epithelial and inflammatory cells [44], *Pseudomonas aeruginosa*-induced pneumonia mice [45], and patients with bronchopulmonary dysplasia [46]. Additionally, Balli et al. reported that up-regulation of FOXM1 enhances radiation-induced pneumonitis and pulmonary fibrosis [47]. Xia et al. revealed that down-regulation of FOXM1 exacerbates lung remodelling and reduces lung function in a mouse model of BPD [46]. Zhu et al. reported that silencing of FOXM1 reverses the inhibitory effects of CRNDE on apoptosis and inflammation as well as the positive impact of CRNDE on cell viability in an LPS-induced cell model of pneumonia [29]. Consistent with the expression trends found in prior studies, we observed a high expression of FOXM1 in the serum of patients with pneumonia and LPS-treated WI-38 cells and mice, indicating that FOXM1 is implicated in the development of pneumonia. Moreover, FOXM1 can act as a downstream target of many miRNAs in lung cancers, such as miR-149 [48], miR-134 [49], miR-509-5p [50], miR-361-5p [51] and miR-145 [52]. Here, we confirmed that FOXM1 is targeted by miR-370-3p and inversely regulated by miR-370-3p. Based on these results, we deduced that miR-370-3p ameliorated LPS-induced injury by targeting FOXM1. Furthermore, we found that overexpression of FOXM1 reversed the protective effects of KCNQ1OT1 silencing in both LPS-treated WI-38 cells and mice. Taken together, we concluded that si-KCNQ1OT1 might serve as a competitive endogenous RNA to regulate the expression of FOXM1 by sponging miR-370-3p, thereby alleviating injury from pneumonia. Our results further confirmed the key role of the KCNQ1OT1/miR-370-3p/FOXM1 axis in the progression of pneumonia, providing new ideas for its clinical treatment.

In summary, lncRNA KCNQ1OT1 was up-regulated in the serum of patients with pneumonia and in LPS-treated WI-38 cells and mice. Silencing of KCNQ1OT1 competitively bound with miR-370-3p regulated FOXM1 expression, which consequently mitigated the LPS-caused injury in WI-38 cells and mice (Additional file 1). Our findings may assist with understanding the function of KCNQ1OT1 in pneumonia and provide potential therapeutic strategies for pneumonia.

## Supplementary Information

**Additional file 1**. A flowchart of the regulatory mechanism of KCNQ1OT1/miR-370-3p/FOXM1 axis in pneumonia.

**Additional file 2.** The original images of western blotting analysis.

## Data Availability

All data in the manuscript is available through the responsible corresponding author.

## References

[1] Mattila JT, Fine MJ, Limper AH, Murray PR, Chen BB, Lin PL (2014) Pneumonia. Treatment and diagnosis. Ann Am Thorac Soc 11 Suppl 4:S189–192. doi:10.1513/AnnalsATS.201401-027PL 10.1513/AnnalsATS.201401-027PL PMC5473649 25148424

[2] Watkins RR, Lemonovich TL (2011). Diagnosis and management of community-acquired pneumonia in adults. Am Fam Phys.

[3] Calina D, Rosu L, Rosu A, Ianoi G, Gofita E (2016). Etiological diagnosis and pharmacotherapeutic management of parapneumonic pleurisy. Farmacia.

[4] Lassi ZS, Kumar R, Das JK, Salam RA, Bhutta ZA (2014). Antibiotic therapy versus no antibiotic therapy for children aged two to 59 months with WHO-defined non-severe pneumonia and wheeze. Cochrane Database Syst Rev.

[5] Kolek V. Community pneumonia - fundamentals of diagnosing and treatment. Vnitr Lek. 63(7–8):514–17. 28933177

[6] Kolek V, Jakubec P, Losse S (2017). Diagnostics and treatment of community-acquired pneumonia—simplicity is the key to success. Vnitr Lek.

[7] Plioplys AV, Kasnicka I (2011). Nebulized tobramycin: prevention of pneumonias in patients with severe cerebral palsy. J Pediatr Rehabil Med.

[8] Cilloniz C, Torres A, Niederman M, van der Eerden M, Chalmers J, Welte T, Blasi F (2016). Community-acquired pneumonia related to intracellular pathogens. Intensive Care Med.

[9] Mandell LA (2015). Community-acquired pneumonia: an overview. Postgrad Med.

[10] Ungureanu A, Zlatian O, Mitroi G, Drocaş A, Ţîrcă T, Călina D, Dehelean C, Docea AO, Izotov BN, Rakitskii VN, Cioboată R, Spandidos DA, Tsatsakis AM, Găman A (2017). Staphylococcus aureus colonisation in patients from a primary regional hospital. Mol Med Rep.

[11] Zlatian O, Balasoiu AT, Balasoiu M, Cristea O, Docea AO, Mitrut R, Spandidos DA, Tsatsakis AM, Bancescu G, Calina D (2018). Antimicrobial resistance in bacterial pathogens among hospitalised patients with severe invasive infections. Exp Ther Med.

[12] Tanase A, Colita A, Ianosi G, Neagoe D, Branisteanu DE, Calina D, Docea AO, Tsatsakis A, Ianosi SL (2016). Rare case of disseminated fusariosis in a young patient with graft vs. host disease following an allogeneic transplant. Exp Ther Med.

[13] Calina D, Docea AO, Petrakis D, Egorov AM, Ishmukhametov AA, Gabibov AG, Shtilman MI, Kostoff R, Carvalho F, Vinceti M, Spandidos DA, Tsatsakis A (2020). Towards effective COVID-19 vaccines: updates, perspectives and challenges (Review). Int J Mol Med.

[14] Ernst C, Morton CC (2013). Identification and function of long non-coding RNA. Front Cell Neurosci.

[15] Ponting CP, Oliver PL, Reik W (2009). Evolution and functions of long noncoding RNAs. Cell.

[16] Zhou Z, Zhu Y, Gao G, Zhang Y (2019). Long noncoding RNA SNHG16 targets miR-146a-5p/CCL5 to regulate LPS-induced WI-38 cell apoptosis and inflammation in acute pneumonia. Life Sci.

[17] Zhang Y, Zhu Y, Gao G, Zhou Z (2019). Knockdown XIST alleviates LPS-induced WI-38 cell apoptosis and inflammation injury via targeting miR-370-3p/TLR4 in acute pneumonia. Cell Biochem Funct.

[18] Nong W (2019). Long non-coding RNA NEAT1/miR-193a-3p regulates LPS-induced apoptosis and inflammatory injury in WI-38 cells through TLR4/NF-kappaB signaling. Am J Transl Res.

[19] Kanduri C (2011). Kcnq1ot1: a chromatin regulatory RNA. Semin Cell Dev Biol.

[20] Kang Y, Jia Y, Wang Q, Zhao Q, Song M, Ni R, Wang J (2019). Long noncoding RNA KCNQ1OT1 promotes the progression of non-small cell lung cancer via regulating miR-204-5p/ATG3 axis. Onco Targets Ther.

[21] Jiang X, Yu M, Zhu T, Lou L, Chen X, Li Q, Wei D, Sun R (2020). Kcnq1ot1/miR-381-3p/ETS2 axis regulates inflammation in mouse models of acute respiratory distress syndrome. Mol Ther Nucleic Acids.

[22] Adams BD, Parsons C, Walker L, Zhang WC, Slack FJ (2017). Targeting noncoding RNAs in disease. J Clin Invest.

[23] Lagos-Quintana M, Rauhut R, Lendeckel W, Tuschl T (2001). Identification of novel genes coding for small expressed RNAs. Science.

[24] Bartel DP (2004). MicroRNAs: genomics, biogenesis, mechanism, and function. Cell.

[25] Zhang L, Dong L, Tang Y, Li M, Zhang M (2020). MiR-146b protects against the inflammation injury in pediatric pneumonia through MyD88/NF-kappaB signaling pathway. Infect Dis (Lond).

[26] Quan B, Zhang H, Xue R (2019). miR-141 alleviates LPS-induced inflammation injury in WI-38 fibroblasts by up-regulation of NOX2. Life Sci.

[27] Zhang J, Mao F, Zhao G, Wang H, Yan X, Zhang Q (2020). Long non-coding RNA SNHG16 promotes lipopolysaccharides-induced acute pneumonia in A549 cells via targeting miR-370-3p/IGF2 axis. Int Immunopharmacol.

[28] Scott JA, Wonodi C, Moisi JC, Deloria-Knoll M, DeLuca AN, Karron RA, Bhat N, Murdoch DR, Crawley J, Levine OS, O'Brien KL, Feikin DR (2012). The definition of pneumonia, the assessment of severity, and clinical standardization in the Pneumonia Etiology Research for Child Health study. Clin Infect Dis.

[29] Zhu-Ge D, Yang YP, Jiang ZJ (2018). Knockdown CRNDE alleviates LPS-induced inflammation injury via FOXM1 in WI-38 cells. Biomed Pharmacother.

[30] Fu L, Zhu P, Qi S, Li C, Zhao K (2018). MicroRNA-92a antagonism attenuates lipopolysaccharide (LPS)-induced pulmonary inflammation and injury in mice through suppressing the PTEN/AKT/NF-kappaB signaling pathway. Biomed Pharmacother.

[31] Parsey MV, Tuder RM, Abraham E (1998). Neutrophils are major contributors to intraparenchymal lung IL-1 beta expression after hemorrhage and endotoxemia. J Immunol.

[32] Reynolds JH, McDonald G, Alton H, Gordon SB (2010). Pneumonia in the immunocompetent patient. Br J Radiol.

[33] Zheng L, Zhang FX, Wang LL, Hu HL, Lian YD (2019). LncRNA KCNQ1OT1 is overexpressed in non-small cell lung cancer and its expression level is related to clinicopathology. Eur Rev Med Pharmacol Sci.

[34] Ren K, Xu R, Huang J, Zhao J, Shi W (2017). Knockdown of long non-coding RNA KCNQ1OT1 depressed chemoresistance to paclitaxel in lung adenocarcinoma. Cancer Chemother Pharmacol.

[35] Wang Y, Yang X, Jiang A, Wang W, Li J, Wen J (2019). Methylation-dependent transcriptional repression of RUNX3 by KCNQ1OT1 regulates mouse cardiac microvascular endothelial cell viability and inflammatory response following myocardial infarction. FASEB J.

[36] Li X, Dai Y, Yan S, Shi Y, Han B, Li J, Cha L, Mu J (2017). Down-regulation of lncRNA KCNQ1OT1 protects against myocardial ischemia/reperfusion injury following acute myocardial infarction. Biochem Biophys Res Commun.

[37] Karreth FA, Pandolfi PP (2013). ceRNA cross-talk in cancer: when ce-bling rivalries go awry. Cancer Discov.

[38] Chen L, Zhou Y, Li H (2018). LncRNA, miRNA and lncRNA-miRNA interaction in viral infection. Virus Res.

[39] Ballantyne MD, McDonald RA, Baker AH (2016). lncRNA/MicroRNA interactions in the vasculature. Clin Pharmacol Ther.

[40] Dong Z, Yang P, Qiu X, Liang S, Guan B, Yang H, Li F, Sun L, Liu H, Zou G, Zhao K (2019). KCNQ1OT1 facilitates progression of non-small-cell lung carcinoma via modulating miRNA-27b-3p/HSP90AA1 axis. J Cell Physiol.

[41] Yu X, Qian X, Sun R, Yang B, Zheng H, Jiang P, Li X (2019). MiR-370-3p targets TLR4 to regulate LPS-induced acute pneumonia in WI-38 cells. Panminerva Med.

[42] Balli D, Ren X, Chou FS, Cross E, Zhang Y, Kalinichenko VV, Kalin TV (2012). Foxm1 transcription factor is required for macrophage migration during lung inflammation and tumor formation. Oncogene.

[43] Kim IM, Zhou Y, Ramakrishna S, Hughes DE, Solway J, Costa RH, Kalinichenko VV (2005). Functional characterization of evolutionarily conserved DNA regions in forkhead box f1 gene locus. J Biol Chem.

[44] Ren X, Shah TA, Ustiyan V, Zhang Y, Shinn J, Chen G, Whitsett JA, Kalin TV, Kalinichenko VV (2013). FOXM1 promotes allergen-induced goblet cell metaplasia and pulmonary inflammation. Mol Cell Biol.

[45] Liu Y, Sadikot RT, Adami GR, Kalinichenko VV, Pendyala S, Natarajan V, Zhao YY, Malik AB (2011). FoxM1 mediates the progenitor function of type II epithelial cells in repairing alveolar injury induced by Pseudomonas aeruginosa. J Exp Med.

[46] Xia H, Ren X, Bolte CS, Ustiyan V, Zhang Y, Shah TA, Kalin TV, Whitsett JA, Kalinichenko VV (2015). Foxm1 regulates resolution of hyperoxic lung injury in newborns. Am J Respir Cell Mol Biol.

[47] Balli D, Ustiyan V, Zhang Y, Wang IC, Masino AJ, Ren X, Whitsett JA, Kalinichenko VV, Kalin TV (2013). Foxm1 transcription factor is required for lung fibrosis and epithelial-to-mesenchymal transition. EMBO J.

[48] Ke Y, Zhao W, Xiong J, Cao R (2013). miR-149 Inhibits Non-Small-Cell Lung Cancer Cells EMT by Targeting FOXM1. Biochem Res Int.

[49] Li J, Wang Y, Luo J, Fu Z, Ying J, Yu Y, Yu W (2012). miR-134 inhibits epithelial to mesenchymal transition by targeting FOXM1 in non-small cell lung cancer cells. FEBS Lett.

[50] Ma N, Zhang W, Qiao C, Luo H, Zhang X, Liu D, Zang S, Zhang L, Bai J (2016). The tumor suppressive role of MiRNA-509-5p by targeting FOXM1 in non-small cell lung cancer. Cell Physiol Biochem.

[51] Hou XW, Sun X, Yu Y, Zhao HM, Yang ZJ, Wang X, Cao XC (2017). miR-361-5p suppresses lung cancer cell lines progression by targeting FOXM1. Neoplasma.

[52] Yuan Y, Haiying G, Zhuo L, Ying L, Xin H (2018). Long non-coding RNA LINC00339 facilitates the tumorigenesis of non-small cell lung cancer by sponging miR-145 through targeting FOXM1. Biomed Pharmacother.

