# Supplementary figures and images for: Silencing of long non-coding RNA KCNQ1OT1 alleviates LPS-induced lung injury by regulating the miR-370-3p/FOXM1 axis in childhood pneumonia
Source: BMC Pulm Med. 2021 Jul 23;21:247. doi: 10.1186/s12890-021-01609-0 (PMC8299180; doi:10.1186/s12890-021-01609-0)

A flowchart of the regulatory mechanism of KCNQ1OT1/miR-370-3p/FOXM1 axis in pneumonia.


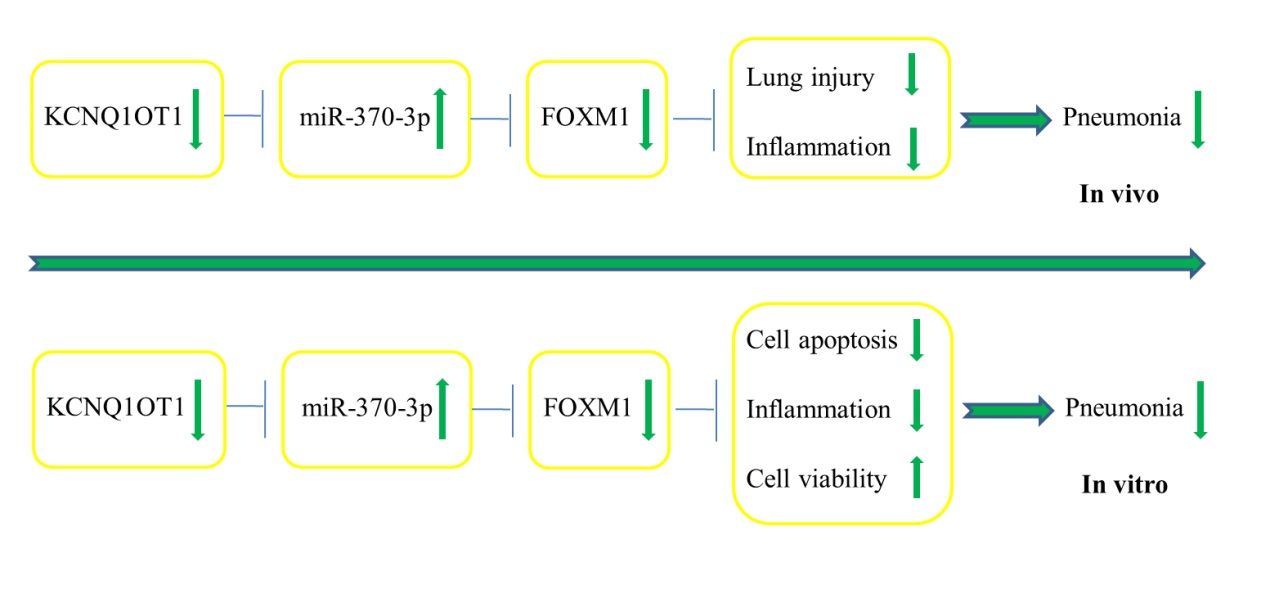

Supplement: Supplementary file 1 — Additional file 1. A flowchart of the regulatory mechanism of KCNQ1OT1/miR-370-3p/FOXM1 axis in pneumonia. [file 12890_2021_1609_MOESM1_ESM.docx]

The original images of western blotting analysis


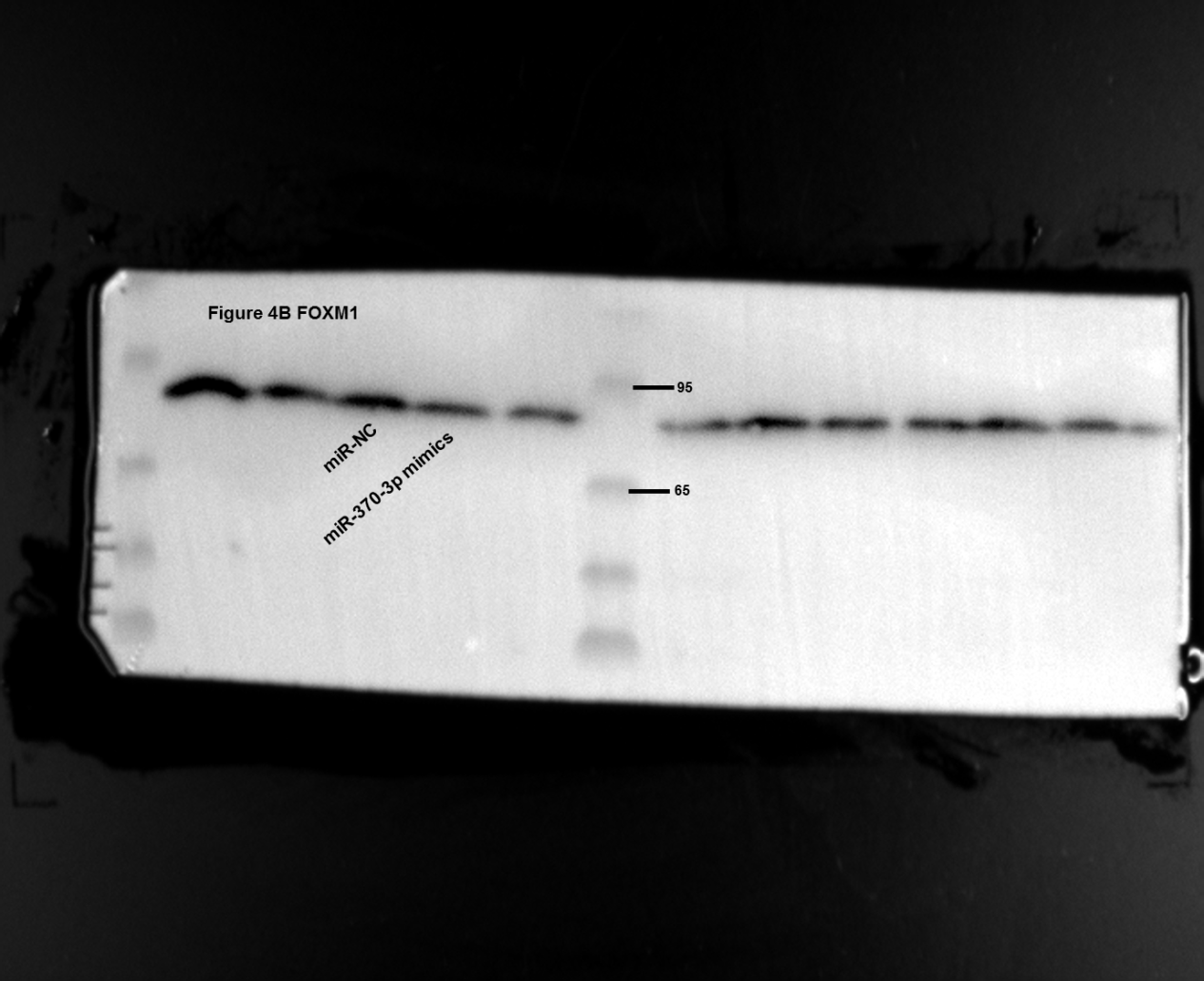


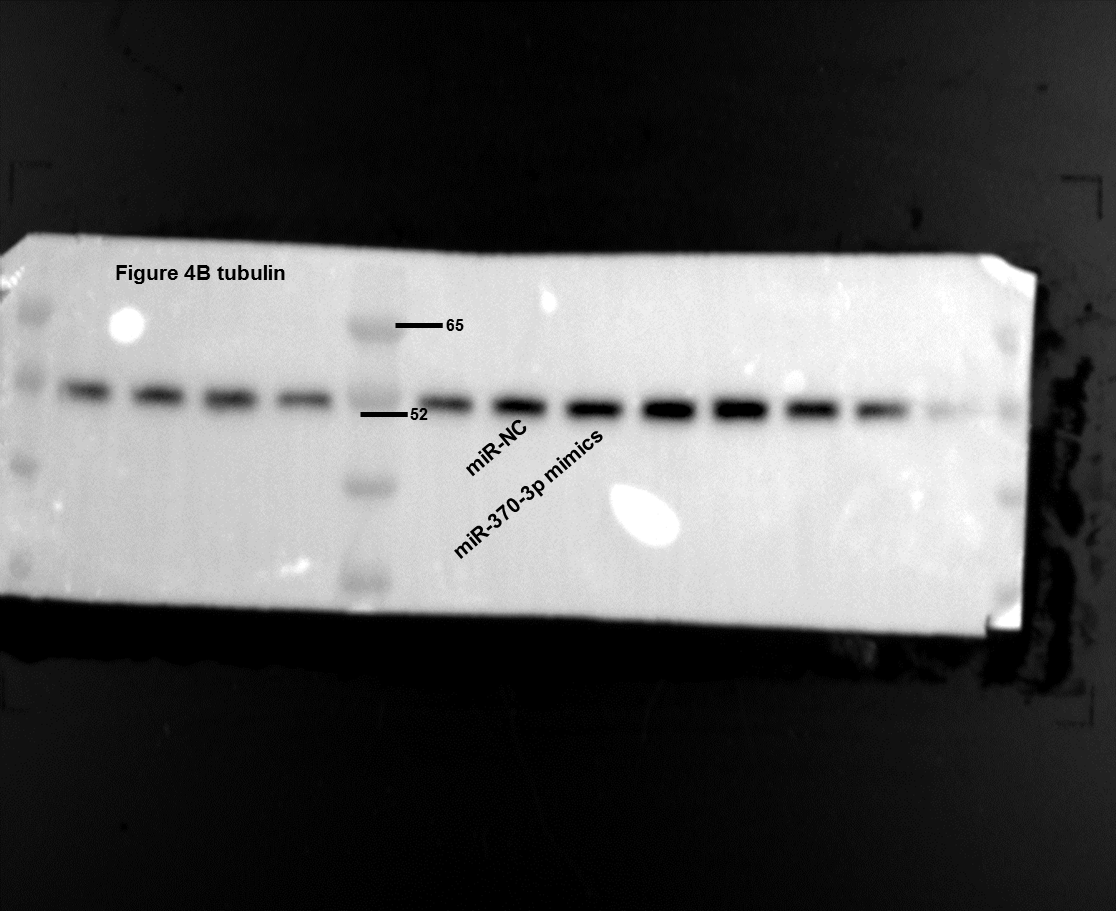


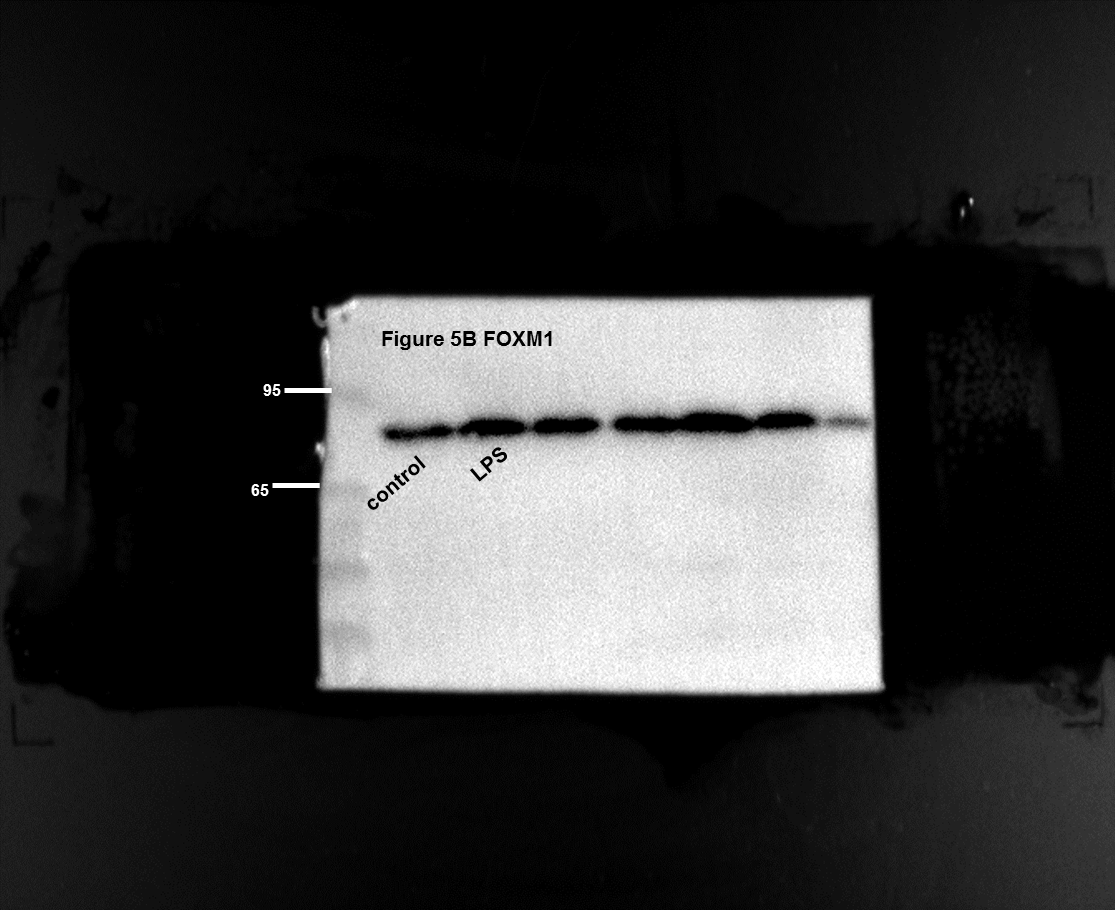


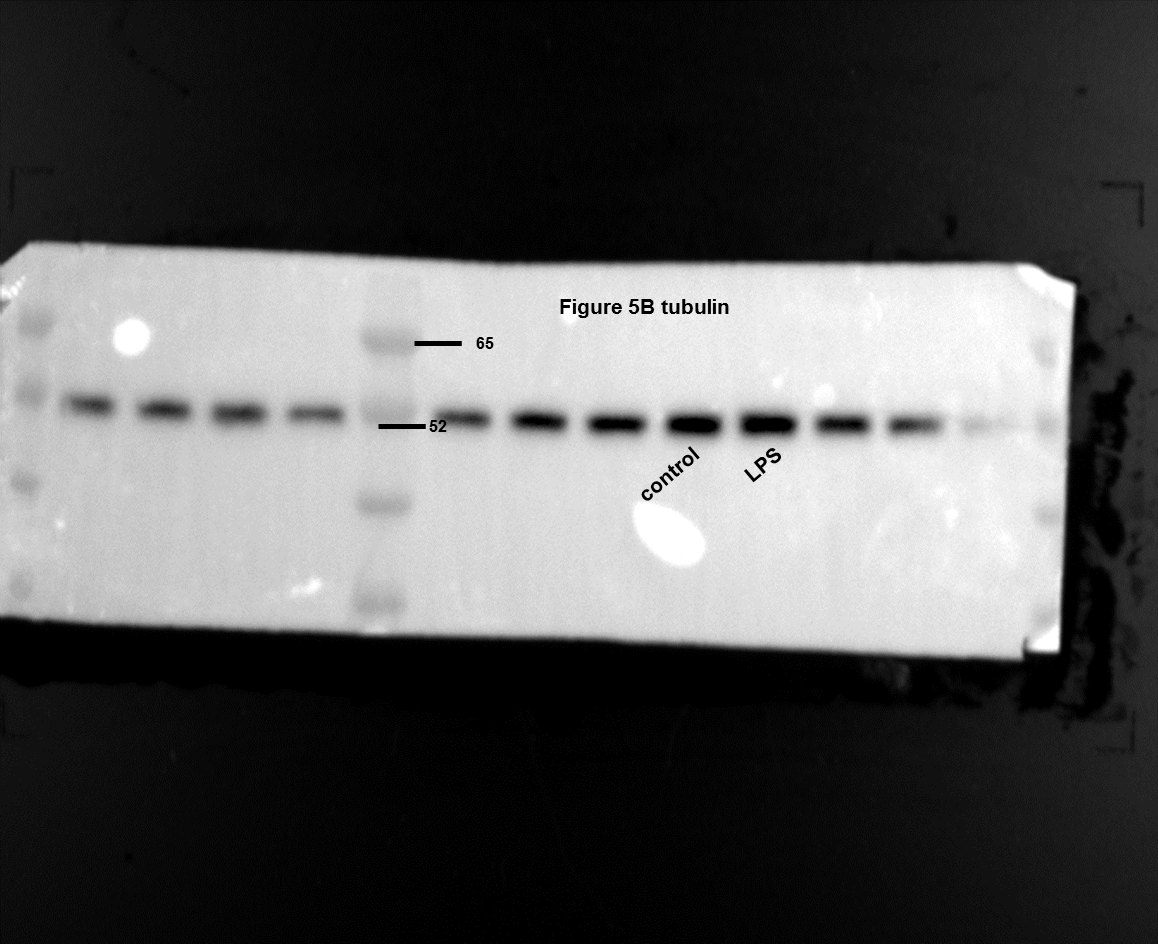


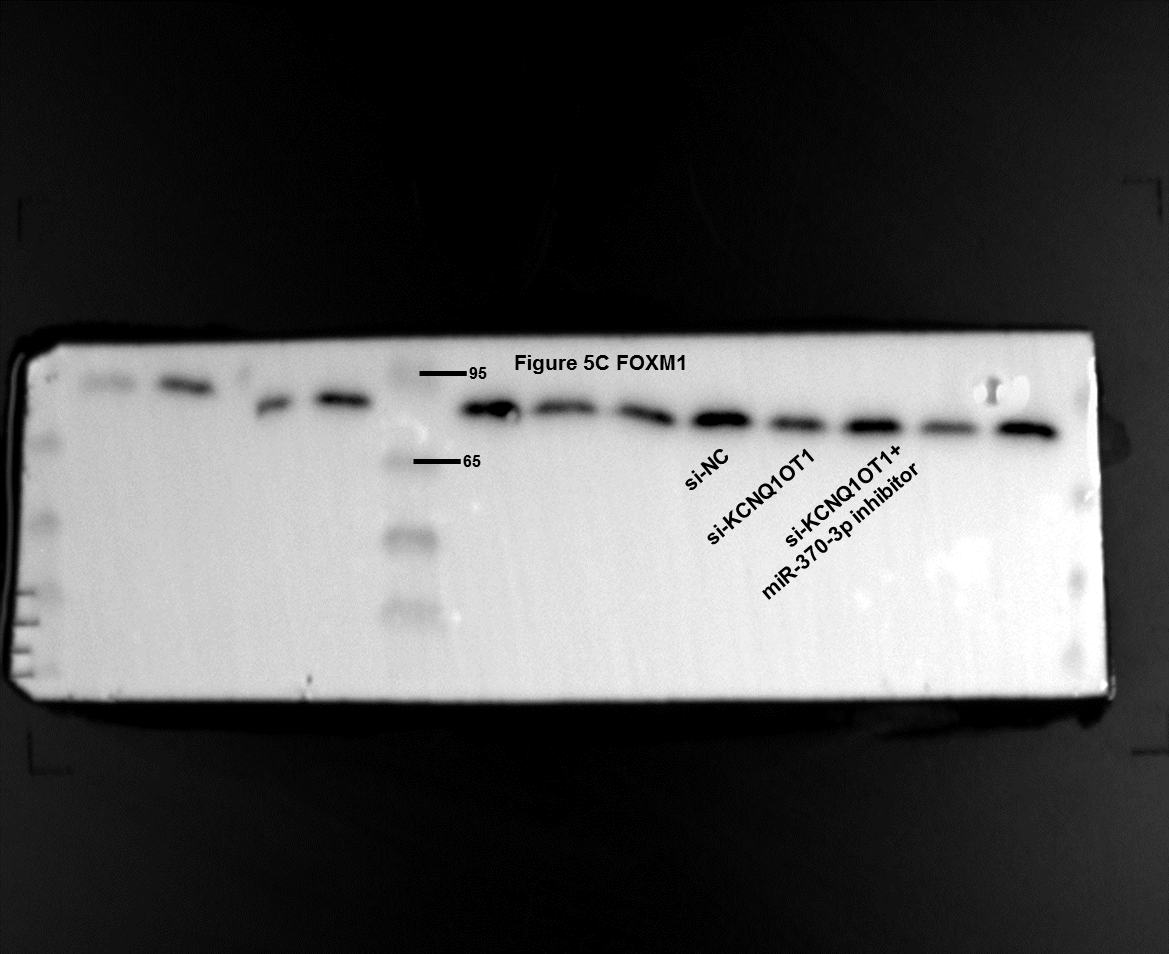


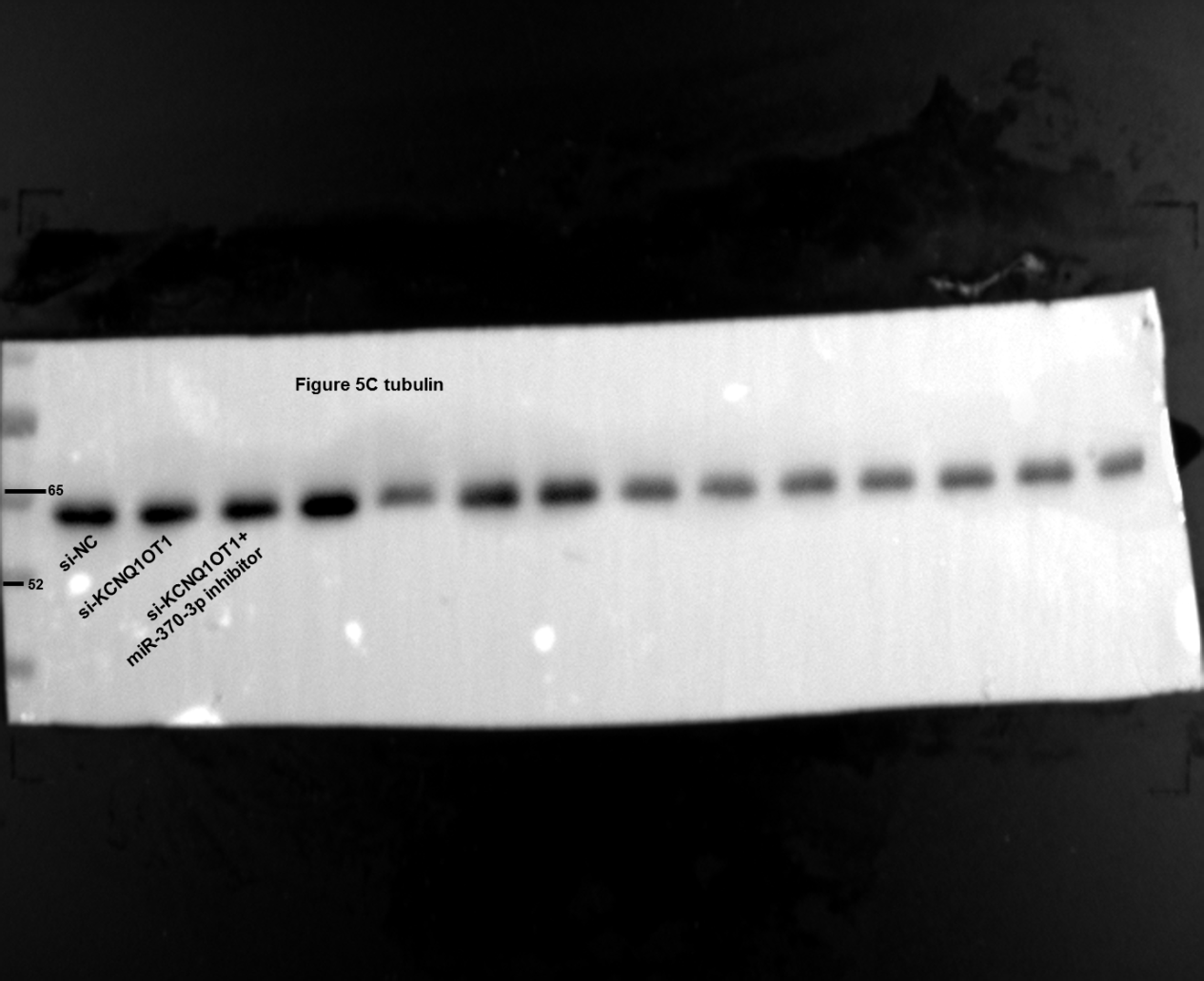

Supplement: Supplementary file 2 — Additional file 2. The original images of western blotting analysis. [file 12890_2021_1609_MOESM2_ESM.docx]
